# Editorial: Animal social behaviour and gut microbiome

**DOI:** 10.3389/fmicb.2023.1210717

**Published:** 2023-08-07

**Authors:** Lifeng Zhu

**Affiliations:** School of Medicine & Holistic Integrative Medicine, Nanjing University of Chinese Medicine, Nanjing, China

**Keywords:** animal social behavior, gut microbial transmission, function, gut-brain-axis, interaction mechanism

## Introduction

As research has progressed, the gut microbiome is widely recognized as playing a pivotal role in host phylogeny, overall health, and even adaptation to extreme environments (Cai et al.; Li et al.; Liu, Han et al.; Yang B. et al.). It has become increasingly clear that the gut microbiome is influenced by a variety of factors, including the host genome, nutrient metabolism, habitat environment, and seasonal dynamics (Wu et al., 2018; Groot et al., 2020; Strasser et al., 2021; Yang et al., 2021; Liu, Chen et al.; Yang X. et al.). Of particular note is the growing interest in the relationship between host behavior and gut microbiota, as studies have demonstrated that host behavioral processes are shaped by the microbiome and represent important predictors of similarities and differences in the composition of the gut microbial communities (Vuong et al., 2017; Nagpal and Cryan, 2021). In recent years, studies involving non-human Primates (NHPs) have been particularly illuminating, with NHPs frequently engaging in diverse social behavior, that has facilitated the transmission of gut microbiota among individuals (Tung et al., 2015; Moeller et al., 2016; Perofsky et al., 2017; Zhu et al., 2020; Pinacho-Guendulain et al., 2022). However, much remains to be further explored regarding the specific effects of individual gut microbiota on host behavioral patterns, as well as the ways in which host behavior shapes the gut microbiome. Notably, research on the relationship between behavior and the gut microbiome in wildlife is very limited at present.

This Research Topic intends to explore recent developments in the field, focusing on, but not limited to: (1) The putative gut microbiome transmission in the wild animal social groups: the quality and quantity level of gut microbiome transmission, (2) The potential reciprocal connections between an animal's social behavior and its gut microbiome: the convergent pattern among different animals, (3) The function of the transmitted microbiome in wild animals, (4) How the microbiomes can affect host behavior, (5) The evolutionary perspective on the relationship between social behavior and the gut microbiome. The Research Topic currently includes 17 original research articles and one review article.

## Microbial transmission shapes gut microbiome

Typically, mammals lack gastrointestinal microbes before birth, requiring each generation to undergo a reassembly of the gut microbiota (Smits et al., 2017). During this period of gut microbiome establishment, both vertical transmission of maternal microbiota (such as breast milk, somatic microbiota, vaginal microbiota, and fecal microbiota) and horizontal transmission of environmental microbiota play vital roles (Dominguez-Bello et al., 2016; Rothschild et al., 2018; Klein-Jöbstl et al., 2019). Since the rapid colonization of the gut microbiome serves to protect the newborn against pathogens in the early stages of life when the immune system is not yet fully developed (Gensollen et al., 2016; Gomez de Agüero et al., 2016). For example, Zhang J. et al. found the importance of maternal fecal microbiota for rapid colonization of the gut microbiota of calves (yak and cattle) at different weeks after birth, and they determined that early establishment of the gut microbiota in the calves was facilitated mainly by maternal fecal microbial transmission. Moreover, social behavior can also promote horizontal transmission of gut microbiota among different species in a shared environment. Zhang T. et al., for instance, discovered that social interactions between goats and pigs in the same pen promoted greater homogenization of both rumen and cecum microbiomes.

The relationship between host social behavior and the microbiome is reciprocal. Social interactions can influence the gut microbial composition, whilst in turn, the gut microbiome can regulate host behavior (Archie and Tung, 2015). For instance, research has demonstrated that the balance of the gut microbiota plays a crucial role in the sexual selection and reproductive behavior of the host (Walsh et al., 2017). In a study of this Research Topic by Yi et al. (2021), it was shown that antibiotic-induced dysbiosis of the gut microbiota in mice reduced the sexual attractiveness of females to males, possibly due to the negative impact of the vertical transmission of dysbiosis of maternal gut microbiota on the immunity and social competence of offspring. Thus, favoring healthy females over those with gut bacteria dysbiosis may facilitate population reproduction (Yi and Cha).

Furthermore, the gut microbiome has been found to affect host reproductive and metabolic endocrine systems (Qi et al., 2021). In patients with central precocious puberty, *Parabacteroides* in the gut were positively correlated with luteinizing hormone-releasing hormone (Li et al., 2021). The interactions between gut microbiota and hormones could contribute to the maintenance of physiological stability, with certain enzymes produced by gut microbiota playing a role in regulating hormone levels (Ridlon and Bajaj, 2015; Ridlon Jason, 2020; Pace and Watnick, 2021). Precocious puberty may have potential effects on animal mating behavior, that the researchers found that the gut microbiota plays a key role in promoting sexual maturation and that gonadotropin-releasing hormone was positively correlated with *Desulfovibrio, Lachnoclostridium, GCA-900066575, Streptococcus, Anaerotruncus*, and *Bifidobacterium*, suggesting that these bacteria may contribute to promoting sexual development (Bo et al., 2021).

## Social behavior—Gut microbiome and host health

Social interactions can enhance the transmission of the social microbiome in social animals, which can fundamentally affect the costs and benefits of group living (Majolo et al., 2008; Sarkar et al., 2020; Yarlagadda et al., 2021). Communal animals can decrease the transmission of pathogens by avoiding diseased individuals or increase the transmission of beneficial microbes through social interactions or fecal consumption, thus promoting the stability and resilience of the gut microbiome and protecting the host from pathogens (Johnson and Burnet, 2016; Stockmaier et al., 2021; Yarlagadda et al., 2021). In this Research Topic, there is research that focuses on this relevant subject. Johnson et al. showed that in the social structure of the non-captive macaque (*Macaca mulatta*) population, the bacteria beneficial to host health (e.g., *Faecalibacterium* with strong anti-inflammatory properties) were more abundant in the gut of sociability individuals, whereas certain potentially pathogenic bacteria (e.g., *Streptococcus*) were more abundant in fewer sociability individuals. Overall, these results highlight the critical link between host health and social behavior-gut microbiome composition.

## Future of animal social behavior and gut microbiome

The relevance, interaction, and importance of host social behavior and the gut microbiome have been reconfirmed in the most recent study on this topic. However, these recent developments simultaneously present us with new inquiries and challenges. It has been demonstrated that intestinal mucosa-associated fungi can influence social behavior by modulating neuroimmune in mice (Leonardi et al., 2022). To date, much of the current research still focuses on elucidating the gut bacterial community, largely neglecting the interactions and potential contribution of other diverse microbial components (e.g., fungi, viruses, protozoa, etc.) on the social behavior-gut microbiome. With the advancement of sequencing technology and bioinformatics analysis, further exploration into diverse gut microbial taxa may become a research hotspot in the future.

Moreover, with the growing number of studies in recent years that have unveiled the mechanisms linking the brain-gut axis to host immune diseases, it has further been evidenced that the brain-gut axis significantly influences host behavior. In particular, the researchers have identified and formulated a more specific microbiota-gut-behavior axis to highlight the critical role in regulating social behavior (Ntranos and Casaccia, 2018). It has become increasingly apparent that these systems are not self-contained, and various elements, including the immune system, bacterial by-products, neurotransmitters, nerve cells, and neuropeptides, are potential mechanisms that mediate normal communication in the brain-gut behavioral axis (Holzer and Farzi, 2014; Erny et al., 2015; O'Mahony et al., 2015; Sampson and Mazmanian, 2015; Hoban et al., 2016). Given that the gut-brain-behavior axis has gradually emerged as crucial in clinical settings (Asadi et al., 2022; Chang et al., 2022; Ribeiro et al., 2022), we argue that it is equally crucial in wildlife conservation. Although there has recently been a substantial body of research in this field, most of these findings are based on clinical and indoor sterile mouse experiments. Thus, more research is still needed in the future to elucidate the components of the brain-gut behavioral axis among wildlife. In addition, interventional treatment of potential brain-gut behavioral axis disorders by probiotics or other microorganisms might further tap into the potential role of the brain-gut behavioral axis in conservation biology (Kesika et al., 2021; Schaub et al., 2022).

The crucial role of maternal vertical transmission of gut microbiota in the early development and lifelong maintenance of the host gut microbiome has been extensively supported by numerous clinical trial results (Vandenplas et al., 2020; Wang et al., 2020; Browne et al., 2022; Xue et al., 2022). Similarly, microbiota transmission in animal social networks is also integral to the overall stability and health of wildlife populations (Colston, 2017; Moeller et al., 2018; Sarkar et al., 2020; Murillo et al., 2022; Zhu, 2022). Antwis et al. (2018) found that social behavior in a population of semi-feral Welsh Mountain ponies could affect the microbial structure of the entire population. Fu et al. (2021) shows that sympatric yaks and pikas on the Tibetan plateau could shift from competition to reciprocity by mutually acquiring beneficial microorganisms through the horizontal transfer of fecal microorganisms. Moreover, chipmunks can acquire beneficial microbes resistant to phytotoxins present in the gut of other insects through the food chain (Yi et al., 2021). Thus, the spread of gut microbiota in animal social networks (intra- or inter-population) warrants additional attention in the future owing to the influence of multiple potential impact mechanisms, such as social behavior (Sarkar et al., 2020), food chains (Moeller et al., 2017) and sympatric species composition (Perofsky et al., 2019; Fu et al., 2021), on animal microbiome transmission.

Despite the initial understanding of the interactions between social behavior and the gut microbiome, the reasons and timing of their relationship, as well as the intergenerational heritability and continuity of social behavior shaping gut microbiome, are still largely unknown (Moeller et al., 2016; Johnson and Foster, 2018; Sherwin et al., 2019). We suggest that multi-animal species analysis may be employed in the future to better elucidate the evolutionary relationship between social behavior and gut microbes. In addition, gaining insight into the endogenous and exogenous mechanisms driving the development of social behaviors, as well as identifying the differences in sociality exhibited by various animal populations over the course of evolution, are critical to unveil the evolutionary relationship between social behaviors and the gut microbiome (Matthews and Tye, 2019; Sherwin et al., 2019).

## Author contributions

The author confirms being the sole contributor of this work and has approved it for publication.
